# Supplementary material for: Evaluation of lay health workers on quality of care in the inpatient setting
Source: PLoS One. 2023 Nov 1;18(11):e0293068. doi: 10.1371/journal.pone.0293068 (PMC10619767; doi:10.1371/journal.pone.0293068)
Supplement: S1 Fig — (DOCX) [file pone.0293068.s001.docx]

**Supporting Information S1 Fig.** Healthcare-associated infections for Units A and B

**Unit A**

Catheter-associated urinary tract infection (CAUTI), central line-associated bloodstream infection (CLABSI), and *C. difficile* rates were not statistically analyzed due to lack of variation in for at least one measurement period.

**Figure a**

*CAUTI Rates at Pre- and Post-Intervention for Unit A*

**
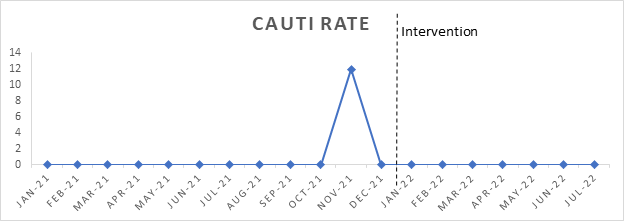
**

**Figure b**

*CLABSII Rates at Pre- and Post-Intervention for Unit A*

**
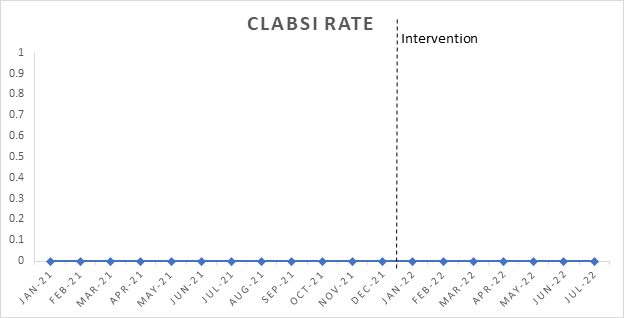
**

**Figure c**

*C. difficile Rates at Pre- and Post-Intervention for Unit A*

**
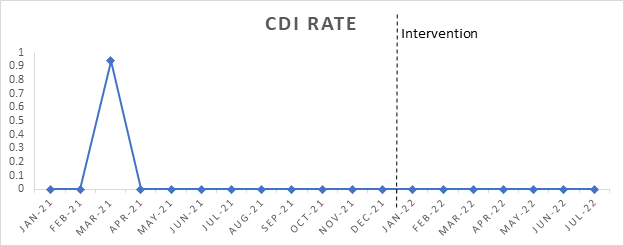
**

**Unit B**

**Figure d**

*CAUTI Rates at Pre- and Post-Intervention for Unit B*

**
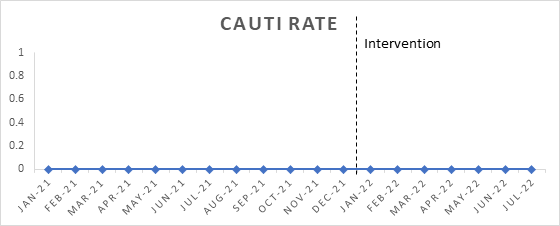
**

**Figure e**

*CLABSI Rates at Pre- and Post-Intervention for Unit B*

**
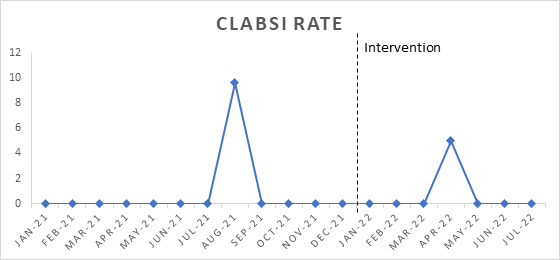
**

**Figure f**

*C. difficile Rates at Pre- and Post-Intervention for Unit B*

**
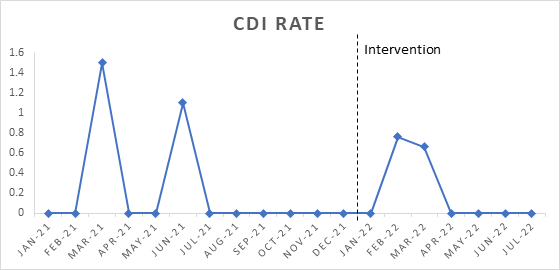
**
